# Supplementary material for: Variation of lightning-ignited wildfire patterns under climate change
Source: Nat Commun. 2023 Feb 10;14:739. doi: 10.1038/s41467-023-36500-5 (PMC9918523; doi:10.1038/s41467-023-36500-5)
Supplement: Supplementary file 1 — Supplementary Information [file 41467_2023_36500_MOESM1_ESM.pdf]

Supplementary information of Variation of lightning-ignited wildfire patterns under climate change

Francisco J. Pérez-Invernón[1,2*], Francisco J. Gordillo-Vázquez[1], Heidi Huntrieser[2] and Patrick Jöckel[2]

[1]Instituto de Astrofísica de Andalucía, Consejo Superior de Investigaciones Cientificas, Glorieta de la Astronomía s/n, Granada, 18008, Andalucía, Spain.
[2] Institut für Physik der Atmosphäre, Deutsche Zentrum für Luft- und Raumfahrt, Münchener Str. 20, Oberpfaffenhofen, 82234, Bayern, Germany.

*Corresponding author(s). E-mail(s): fjpi@iaa.es;
Contributing authors: vazquez@iaa.es; Heidi.Huntrieser@dlr.de; Patrick.Joeckel@dlr.de;

Keywords: lightning, wildfires, climate change, long-continuing-current lightning

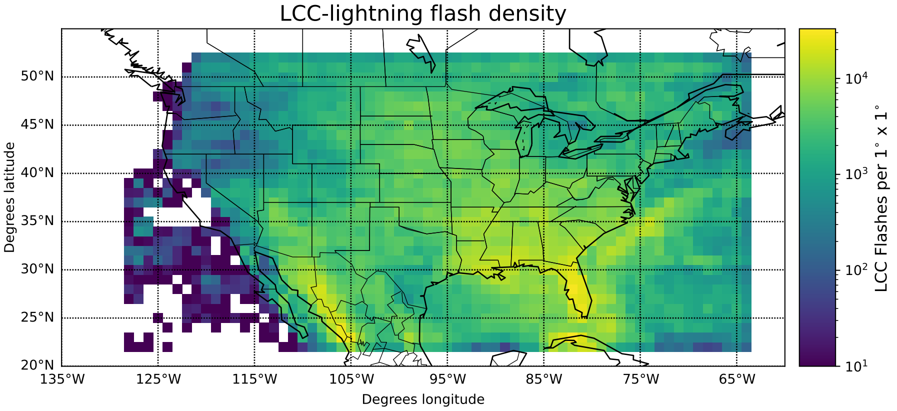

Fig. S1 Long-Continuing-Current (LCC) lightning density from the Geostationary Lightning Mapper (GLM) between 15 May 2018 and 31 August 2018 by applying the classification method developed by Fairman and Bitzer (2022) [1] binned into 1° × 1° grids.

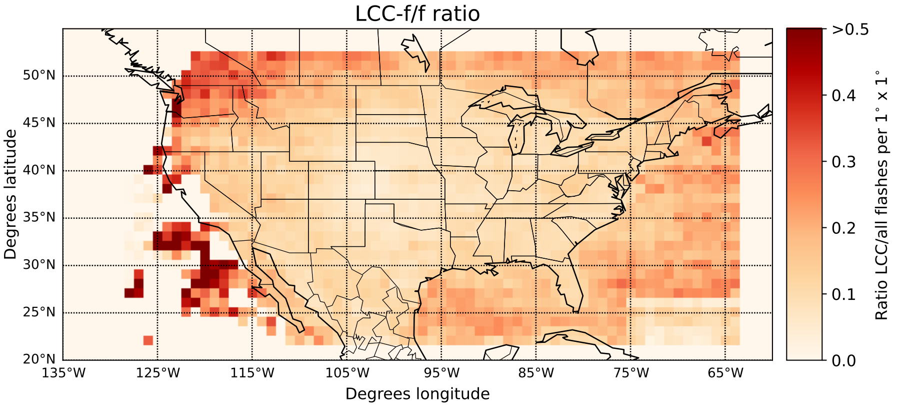

Fig. S2 Spatial distribution of the ratio of Long-Continuing-Current (LCC) lightning to total lightning from GLM between 15 May 2018 and 31 August 2018 by applying the classification method developed by Fairman and Bitzer (2022) [1] binned into 1° × 1° grids.

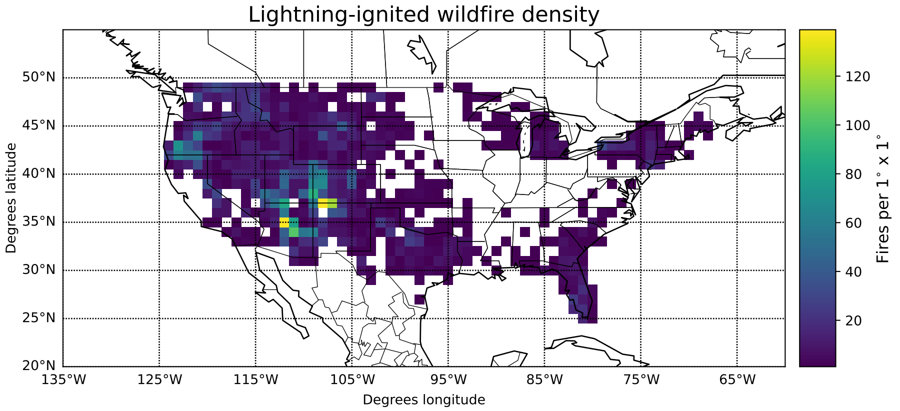

Fig. S3 Lightning-ignited wildfire density over Continental United States between 1 June 2018 and 31 August 2018 reported by Wright et al. (2011) [2] and Short (2021) [3] binned into 1° × 1° grids.

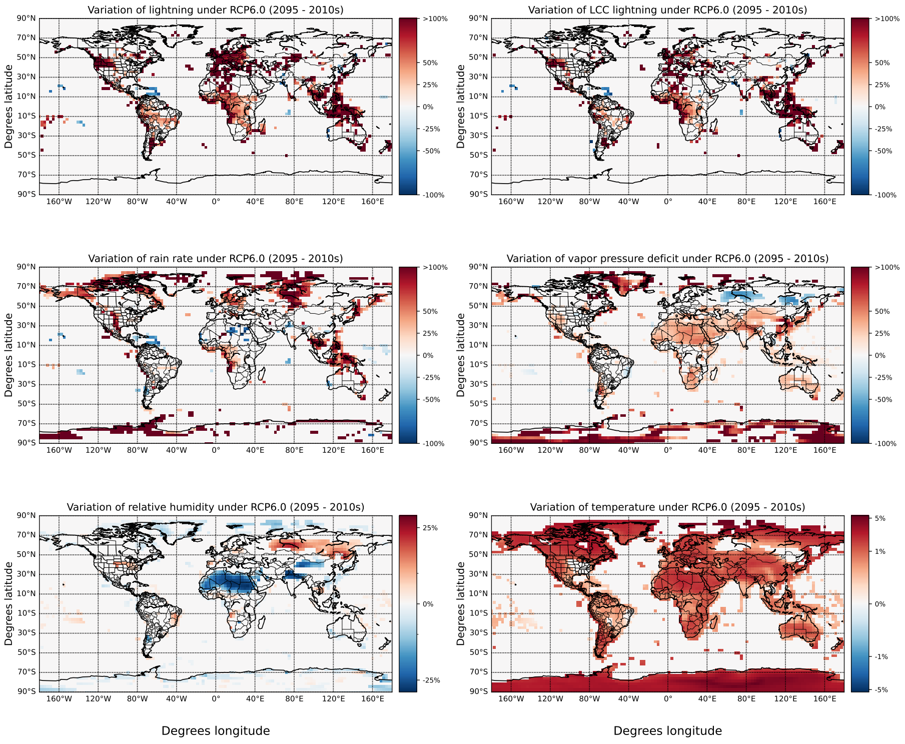

Fig. S4 Annually averaged change of total lightning, Long-Continuing-Current (LCC) lightning, total rain rate, vapor pressure deficit, relative humidity and temperature in each grid cell where the difference is statistically significant under climate change. The values are masked over the ocean. Changes have been calculated from the variations between the periods 2009-2011 and 2091-2095. The color bar have been deliberately saturated at the upper end at 100% due to the high variability of some variables.

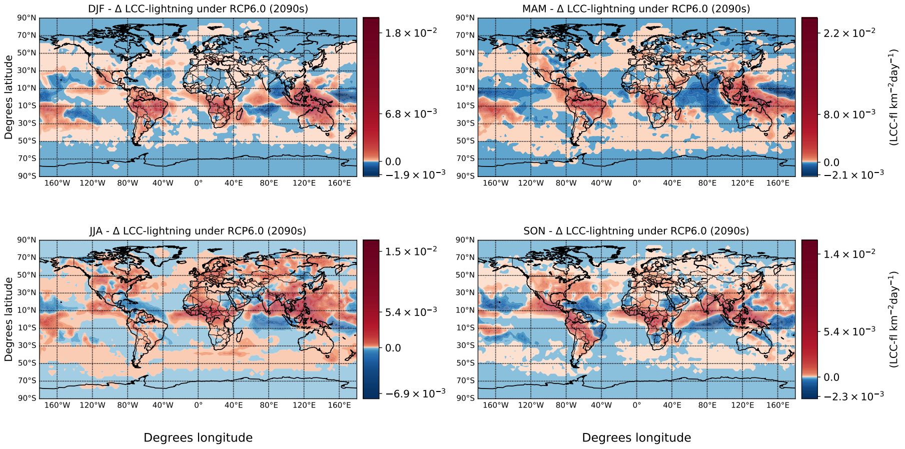

Fig. S5 Seasonally averaged changes of total Long-Continuing-Current (LCC) lightning flash rate between the periods 2009-2011 and 2091-2095. Changes are calculated as the difference between the Representative Concentration Pathway RCP6.0 and present-day simulations. The seasons are December-January-February (DJF), March-April-May (MAM), June-July-August (JJA) and September-October-November (SON).

Supplementary References

[1] Fairman, S.I., Bitzer, P.M.: The detection of continuing current in lightning using the geostationary lightning mapper. J. Geophys. Res. Atmos. **127**(5), 2020–033451 (2022). https://doi.org/10.1029/2020JD033451

[2] Wright, D.K., Glasgow, L.S., McCaughey, W.W., Sutherland, E.K.: Coram experimental forest 15 minute streamflow data. Fort Collins, CO: U.S. Department of Agriculture, Forest Service, Rocky Mountain Research Station (2011). https://doi.org/10.2737/RDS-2011-0019

[3] Short, K.C.: Spatial wildfire occurrence data for the United States, 1992-2018 [fpa_fod_20210617]. 5th Edition. Fort Collins, CO: Forest Service Research Data Archive (2021). https://doi.org/10.2737/RDS-2013-0009.5
